# Reducing ICU blood draws with artificial intelligence

**DOI:** 10.1186/cc11043

**Published:** 2012-03-20

**Authors:** FC Cismondi, AS Fialho, SM Vieira, LA Celi, SR Reti, JM Sousa, SN Finkelstein

**Affiliations:** 1 Massachusetts Institute of Technology, Cambridge, MA, USA; 2 IST, Lisbon, Portugal; 3 BIDMC, Brookline, MA, USA

## Introduction

Recent studies have demonstrated that frequent laboratory testing does not necessarily relate to better outcomes. Our aim is to reduce unnecessary blood draws for ICU laboratory tests by predicting which tests are likely to return as normal or abnormal and therefore influence clinical management around gastrointestinal (GI) bleeding.

## Methods

An artificial intelligence tool, namely fuzzy systems, was applied to 1,092 GI bleed patients extracted from a large ICU database with over 32,000 patients. A classification approach for laboratory test outcome was utilized for a total of seven outcome variables shown in Table 1. The outcome for each test was binarized as normal or abnormal. Input variables included 10 physiological variables such as heart rate, temperature and urine output, as well as further data on transfusions for platelets, red blood cells and plasma.

**Table 1:** Classification results

Outcome	ACC (%)	Sensitivity	Specificity
Calcium	85 ± 2.3	0.88 ± 0.3	0.81 ± 0.1
PTT	86 ± 1.2	0.89 ± 0.1	0.82 ± 0.2
Hematocrit	82 ± 1.6	0.84 ± 0.2	0.78 ± 0.1
Fibrinogen	84 ± 2.8	0.87 ± 0.3	0.80 ± 0.4
Lactate	80 ± 2.2	0.82 ± 0.2	0.77 ± 0.4
Platelets	88 ± 1.3	0.90 ± 0.1	0.85 ± 0.2
Hemoglobin	84 ± 3.1	0.85 ± 0.3	0.81 ± 0.2

ACC, accuracy of classification.

## Results

Classification accuracy of greater than 80% was achieved for all of the seven outcome variables (Table 1). Sensitivity and specificity were satisfactory for all the outcomes. Input variables frequently selected as most predictive of normal or abnormal results include urine output and red blood cell transfusion.

## Conclusion

Reducing frequent laboratory testing, and potential phlebotomy complications, is a major concern in critical care medicine. If one could predict in advance whether a laboratory test would be normal or abnormal then that particular laboratory test may not be ordered, and thereby reducing potential complications and costs. In this work we present an artificial intelligence method for the classifying the likelihood of a blood test being normal or abnormal. Our results show acceptable classification accuracy both in terms of sensitivity and specificity.

